# Concurrent targeting of BMI1 and CDK4/6 abrogates tumor growth *in vitro* and *in vivo*

**DOI:** 10.1038/s41598-019-50140-0

**Published:** 2019-09-23

**Authors:** Ramesh Elango, Radhakrishnan Vishnubalaji, Muthurangan Manikandan, Sarah Ibrahim Binhamdan, Abdul-Aziz Siyal, Yasser A. Alshawakir, Musaad Alfayez, Abdullah Aldahmash, Nehad M. Alajez

**Affiliations:** 1 Cancer Research Center, Qatar Biomedical Research Institute (QBRI), Hamad Bin Khalifa University (HBKU), Qatar Foundation (QF), PO Box 34110, Doha, Qatar; 2 Stem Cell Unit, Department of Anatomy, College of Medicine, King Saud University, Riyadh, Saudi Arabia; 3 Experimental Surgery and Animal Lab, College of Medicine, King Saud University, Riyadh, Saudi Arabia; 4 Prince Naif Health Research Center, King Saud University, Riyadh, Saudi Arabia

**Keywords:** Cancer therapy, Molecular biology

## Abstract

Despite recent advances in cancer management and therapy, resistance to cytotoxic medications remains a major clinical challenge; hence, combination-based anti-cancer treatment regimens are currently gaining momentum. PTC-209 reduced BMI1 protein expression, while palbociclib inhibited CDK4, Rb, and pRb^Ser795^ protein expression in MDA-MB-231 cells. PTC-209 and palbociclib exhibited dose-dependent cytotoxic effects against MDA-MB-231 (breast), HCT116 (colon), and PC-3 (prostate) models, which was more profound in the combination group. Transcriptome and pathway analyses revealed inhibition of insulin signaling, focal adhesion, DNA damage response, and Wnt/pluripotency signaling pathways as well as cell proliferation, and cellular movement functional categories by PTC-209. Transcriptome and pathway analyses revealed palbociclib to mainly affect cell cycle progression and survival. Upstream analysis identified several networks affected by PTC-209 (EZH2, IFNB1, TRIB3, EGFR, SREBF1, IL1A, ERG, TGFB1, MAX, MNT) and palbociclib (RABL6, MITF, RARA, TAL1, AREG, E2F3, FOXM1, ESR1, ERBB2, and E2F). PTC-209 and palbociclib reduced colony and sphere formation, cell migration, and cell viability, which was further enhanced in the combination group. Concordantly, combination of PTC-209 and palbociclib exhibited more profound effects on MDA-MB-231 tumor formation *in vivo*. Our data suggest concurrent targeting of BMI1 and CDK4/CDK6 might provide novel therapeutic opportunity for breast, colon, and prostate cancer.

## Introduction

Malignant transformation is a complex process manifested by the acquisition of sequential genetic alteration overtime^1,2^. Single cell analysis has revolutionized our understanding of tumorigeneses, revealing the complex nature of tumor mass, where different tumor cells within the tumor bulk appears to harbor distinct molecular signatures^3^. This phenomenon has a critical significance during tumor progression and the response to therapeutic interventions^4^. Therefore, achieving pathological complete response is oftentimes challenging, possibly due to the presence of resistant clones, which could be enriched during the course of conventional chemotherapy^5,6^. Tumor initiating cells (TICs), which are believed to initiate and recapitulate the tumor hierarchy, appear to play a pivotal role in tumor growth, proliferation, invasion, drug resistance, distant metastasis and relapse of numerous cancer types^7^, making them an attractive candidate for targeted cancer therapies^8^. TICs have been identified in multiple cancer types including breast^9^, prostate^10^, colorectal^11^, lung^12^, ovarian^13^ and cervical cancers^14^.

B Lymphoma Mo-MLV Insertion Region 1 Homolog (BMI1), the core component of the polycomp repressive complex 1 (PRC1), plays an important role during normal cell development^15^ and malignant transformation^16^. BMI1 is implicated in TICs self-renewal through the regulation of genes important for cell cycle control, DNA damage response and stem cell fate decisions, as well as regulation of pro-survival genes and inhibition of cellular senescence in multiple cancer models^17,18^. BMI1 also controls important stages in cancer progression, including invasion and metastasis by modulating epithelial-mesenchymal transition (EMT)^16^ and drug resistance^19^. We previously shown BMI1 to endow cancer cells with radiation therapy resistance properties through the repression of P53-mediated apoptosis^20^. Kreso *et al*. recently identified PTC-209 as small-molecule inhibitor targeting BMI1, which was proven efficacious in reducing TICs in colorectal cancer *in vitro* and *in vivo*^21^.

Combination therapies are currently gaining momentum for cancer treatment as it allows simultaneous targeting of different genetic pathways^22^. Palbociclib is a highly selective inhibitor of cyclin-dependent kinases 4 and 6 (CDK 4/6), which has been approved by the US Food and Drug Administration (FDA) to treat hormone receptor positive (HR+)/human epidermal growth factor receptor 2 negative (HER2−) breast cancer. Data from a randomized phase II study suggested combination of palbociclib and letrozole to significantly improve progression-free survival in advanced estrogen receptor-positive (ER+) HER2− breast cancer patients^23^. Subsequent phase III clinical trial revealed longer overall survival (OS) for HR+/HER2− advanced breast cancer patients, who progressed or relapsed during previous endocrine therapy, when treated with combination of palbociclib and fulvestrant (median OS = 34.9 months) compared to the placebo-fulvestrant group (median OS = 28.0 months)^24^, setting a foundation for the development of future palbociclib-based combination treatment regimens. In current study, we provide the first experimental evidence on the therapeutic efficacy of combination of palbociclib and PTC-209 against a panel of cancer models (triple negative breast cancer (TNBC), colon, and prostate) and elucidated the molecular mechanisms by which concurrent targeting of TICs and cell cycle progression concurred tumor development.

## Materials and Methods

### Drug preparation

PTC-209 and palbociclib HCL small molecule inhibitors were purchased from Selleckchem (Houston, TX, USA). Inhibitors were dissolved in dimethyl sulfoxide (DMSO) (Sigma Aldrich, St. Louis, MO, USA) at a stock concentration of 10 mM and were stored in aliquots at −20 °C. Further dilutions were made in DMEM at the time of experiment to achieve final concentrations ranging between 0.04 μM and 10 μM.

### Maintenance of cancer cell lines

Human TNBC (MDA-MB-231), prostate (PC-3) and colon (HCT116) cancer cell lines were obtained from the American Type Culture Collection (ATCC, Manassas, VA, USA). Cancer cells were cultured in Dulbecco’s Modified Eagle Medium (DMEM) supplemented with 10% fetal bovine serum (FBS) and 1% penicillin/streptomycin (Pen-Strep), all were purchased from Thermo Scientific (Thermo Scientific, Rockford, IL, USA). Cells were cultured as an adherent monolayer at 37 °C under 5% CO_2_ in a humidified incubator.

### Measurement of cell viability

Cell viability was measured using alamarBlue (Thermo Scientific, Rockford, IL, USA) assay according to the manufacturer’s recommendations as described before^25^. In brief, 4 × 10^3^ cancer cells were seeded in a flat-bottom 96-well plates in the presence of PTC-209 or palbociclib (0.07–10 μM) as single agents or in combination (0.07–10 μM). On day 4, 20 µl of alamarBlue was added to each well at a final concentration of 10% and then plates were incubated in the dark at 37 °C for 2 hrs. Quantification of fluorescent signal intensity was performed using a BioTek Synergy II (BioTek Inc., Winooski, VT, USA) fluorescent plate reader at an excitation and emission wavelengths of 540 nM and 590 nM, respectively. Cell viability of cancer cell lines is presented as percent viability of treated cells compared to DMSO vehicle-treated control cells. PTC-209, palbociclib and combinational (PTC-209 plus palbociclib) were used at 5 μM concentration in subsequent experiments, unless indicated otherwise. Each experiment had a minimum of three replicates and was conducted at least twice.

### Colony formation assay

The colony forming ability of cancer cells treated with the indicated small molecule inhibitor was determined using clonogenic assay as described before^26^. Briefly, 4 × 10^4^ cells were seeded in 2 ml culture medium in 12-well flat-bottom tissue culture plate, followed by subsequent serial dilution (1:1 to 1:32) in the presence of PTC-209 (5.0 μM), palbociclib (5.0 μM), or combination of PTC-209 and palbociclib (5.0 μM each) compared to DMSO vehicle control. The media was changed with fresh media supplemented with the appropriate inhibitor every 3–4 days. On day 10, the plates were washed and stained with Diff-Quik Staining Kit (Siemens Healthcare Diagnostics, USA), and were subsequently scanned and the number of colonies were observed under inverted microscope.

### Sphere formation assay

MDA-MB-231 cells were trypsinized, counted, re-suspended in culture media, and were plated in 60 mm low cell binding dishes (Nunc, Thermo Scientific, Rockford, IL, USA) at 3 × 10^4^ cells/well as we previously described^27^ in the presence of PTC-209 (5.0 μM), palbociclib (5.0 μM), or combination of both inhibitors (5.0 μM each). Cells treated with DMSO were used as experimental control. On day 13, the size and number of multicellular tumor spheroids in each well were counted using an inverted microscope (Axio Observer-A1, Carl Zeiss, Germany) at 10X magnification.

### Scratch assay

MDA-MB-231 cell migration was assessed using the scratch assay as described before^28^. Cells were seeded in 12-well plates and once they reached confluence, cells were treated with the indicated inhibitors for overnight. Vertical wounds were subsequently created using a sterile 200-µl pipette tip. To eliminate cell debris, adherent cells were washed twice with fresh media before supplementation with fresh medium containing the appropriate inhibitor at 5.0 μM compared to cells cultured in the presence of DMSO vehicle control. Cell migration was subsequently monitored and scored at 24 hrs and 48 hrs using phase contrast microscope (Axio Observer-A1, Carl Zeiss, Germany) at 5X magnification.

### Transwell migration assay

A transwell migration system utilizing 8.0 µm pore polyethylene terephthalate (PET, BD Falcon, MA, USA) inserts was used for the migration experiments as we previously described^29^. Briefly, inserts were placed in 12-well notch plate, and 5 × 10^4^ cells were suspended in DMEM supplemented with 1.0% serum and were seeded in the upper chamber, while DMEM supplemented with 10% serum was used as attractant in the lower chamber. Twenty four hours later, non-migrating cells were gently removed using cotton swab, while migrated cells adherent to the lower surface of the membrane were stained with Diff-Quik Staining Kit (Siemens Healthcare Diagnostics, USA). The number of migrated cells was counted in six different random fields using bright-field microscope (Axio Observer-A1, Carl Zeiss, Germany) at 5X magnification.

### Immunoblotting

MDA-MB-231 cells were treated with 5.0 μM of PTC-209 or 5.0 μM of palbociclib and 72 hrs later, cells were harvested and washed twice with cold PBS and were kept on ice for 30 min in radioimmunoprecipitation assay buffer (RIPA) supplemented with halt protease inhibitor cocktail (Pierce Inc., Thermo Scientific, Rockford, IL, USA). Thirty micrograms of total protein were mixed with Laemmli sample buffer and were resolved by Mini-PROTEAN TGX precast gels and then were transferred to polyvinylidene fluoride (PVDF) membrane using Trans-Blot Turbo Blotting System (Bio-Rad, CA, USA) as described earlier^30^. Nonspecific sites were blocked using 5.0% (w/v) skimmed milk in PBS-T (Phosphate-buffered saline: pH 7.4, and 0.1% Tween 20). The transfer membranes were incubated overnight at 4 °C with the monoclonal anti-BMI1 antibody (1:1000, Cell Signaling Technology, USA), rabbit polyclonal anti-pRb (S795; 1:500, Abcam, USA), mouse monoclonal anti-Rb (1:500, Abcam, USA), mouse monoclonal anti-CDK4 (1:1000; Thermo Scientific, Rockford, IL, USA) anti-GAPDH (1:1000; Thermo Scientific, Rockford, IL, USA) in 5% non-fat dry milk in PBS-T. Subsequently, the blots were washed thrice (5 min each) with PBS-T and then incubated with goat anti-mouse IgG (1:3000) conjugated to horseradish peroxidase (HRP) for 2 hrs at room temperature followed by three 5-minute washes in PBS-T. Positive immunoreactive bands were detected using western sure chemiluminescent substrate (LI-COR, Lincoln, NE, USA).

### Total RNA isolation, cDNA synthesis, and quantitative real-time PCR (qRT-PCR)

Seventy two hour post inhibitor treatment (5.0 μM), total RNA was isolated from treated and control cancer cells using total RNA purification kit (Norgen Biotek Corp., ON, Canada) according to the manufacturer’s protocol. The concentrations and purity of extracted RNA was measured using NanoDrop 2000 (Thermo Fisher Scientific, DE, USA). Subsequently, 500 ng of total RNA was reverse transcribed using high capacity cDNA reverse transcript kit (Applied Biosystem, CA, USA). Gene expression was quantified using Fast SYBR™ green master mix and a ViiA7 Real-Time PCR system (Applied Biosystems, CA, USA) according to the manufacturer’s protocol. The relative fold change in mRNA expression was calculated using the 2^−ΔΔCt^ method^31^, where the average of ΔCt values for the amplicon of interest were normalized to that of an endogenous gene (ACTNB), compared with control samples. The primer sequences used in this study are listed in Table 1.

**Table 1:** SYBR green primer sequences used in current study.

No	Name	Forward Sequence	Reverse Sequence
1	ACTNB	5′TGTGCCCATCTACGAGGGGTATGC3′	5′GGTACATGGTGGTGCCGCCAGACA3′
3	IGF2BP2	5′CTGGCCGTGTTCCGGGAGAA3′	5′TTCCTGTTGGCAGGGAGTCCTGG3′
4	GADD45A	5′AGCAGAAGACCGAAAGGATGG3′	5′TGACTCAGGGCTTTGCTGAG3′
5	GADD45B	5′AACGACATCAACATCGTGCG3′	5′GTGTGAGGGTTCGTGACCAG3′
6	RAD51	5′GGGGCAAGCGAGTAGAGAAG3′	5′GCATTGCCATTACTCGGTCC3′
7	PLAU	5′ACTCCAAAGGCAGCAATGAAC3′	5′GTGCTGCCCTCCGAATTTCT3′
9	E2F1	5′AACTGACCATCAGTACCTGGC3′	5′GGGATTTCACACCTTTTCCTGG3′
10	E2F2	5′AGGAGCAGACAGTGATTGCC3′	5′GGTTGTCCTCAGTCCTGTCG3′
11	CCNB1	5′GACAACTTGAGGAAGAGCAAGC3′	5′TTTCCAGTGACTTCCCGACC3′
12	IL-6	5′CGAGCCCACCGGGAACGAAA3′	5′GGACCGAAGGCGCTTGTGGAG3′
13	BCL2	5′AGATTGATGGGATCGTTGCCT3′	5′AGTCTACTTCCTCTGTGATGTTGT3′
14	BIRC5	5′AGCCAAGAACAAAATTGCAAAGG3′	5′CGCACTTTCTCCGCAGTTTC3′

### Cell cycle analysis using flow cytometry

MDA-MB-231, PC-3, and HCT116 cancer cell lines were treated with each inhibitor as single agent or in combination at the indicated dose for 72 hrs. Cells were then trypsinized, washed, and re-suspended at 1 × 10^6^ cells/ml in FACS buffer. Cells were then centrifuged, fixed, and permeablized by slow addition of ice-cold 70% ethanol to the pellet under constant vortexing and then were washed with PBS. Cell pellets were incubated in FACS buffer containing RNAse A for 30 minutes on ice. The cells were centrifuged, and the pellets were resuspended in propidium iodide (PI) staining solution and then subjected to cell cycle analysis using navios flow cytometer (Beckman-Coulter Quanta, CA, USA) at FL3 (605–635) channel, with an excitation maximum at 536 nm and fluorescence emission maximum at 617 nm.

### Detection of apoptosis/necrosis using fluorescence microscopy

The acridine orange and ethidium bromide (AO/EB) fluorescence staining method was used to assess apoptosis/necrosis in cells after exposure to 5.0 μM of PTC-209, palbociclib, or combination of both. After 72 hrs of treatment, control and treated cancer cells were washed twice with PBS and subsequently stained with dual fluorescent staining solution containing 100 μg/ml AO and 100 μg/ml EB (AO/EB, Sigma Aldrich, St. Louis, MO, USA) for two minutes; subsequently, the cells were observed and imaged under Nikon Eclipse Ti2 fluorescence microscope (Nikon, Tokyo, Japan). The differential uptake of AO/EB allows the identification of viable and non-viable cells. Principally, Acridine Orange was used to visualize the number of cells undergone apoptosis, while EB positive cells indicated necrotic cells.

### Gene expression profiling using microarray and pathway analysis

One hundred nanograms of total RNA was labeled using the low input Quick Amp Labeling Kit (Agilent Technologies, CA, USA) and then hybridized to the Agilent Human SurePrint G3 Human GE 8 × 60 k microarray chip as we previously described^32^. The extracted data were normalized and analyzed using GeneSpring 13.0 software (Agilent Technologies). Pathway analyses were performed using the single experiment pathway analysis feature in GeneSpring 13.0 as described earlier^20^. Two-fold cutoff and p < 0.05 (Benjamini-Hochberg multiple testing corrected) were used to determine significantly changed transcripts. Ingenuity pathway and functional annotation analyses were conducted using Ingenuity pathway (Ingenuity Systems, http://www.ingenuity.com)^33,34^. Differentially expressed genes exhibiting ≥ 2 ≤ and corrected P value < 0.05 were subjected to core analysis using the human database. Enriched networks categories were algorithmically generated based on their connectivity and ranked according to the Z score. A Z score ≤ or ≥2.0 was considered significant.

### ***In vivo*** xenograft assay

*In vivo* experiments were approved by the Institutional Animal Care Committee, King Saud University and were conducted as we previously described^26,35^. All methods were performed in accordance with the relevant guidelines and regulations. Briefly, four to six weeks old, nude mice (bred at the Experimental Surgery and Animal Laboratory, King Khalid University Hospital) were utilized for the xenograft experiments and were housed under conditions of a 12 hour light/dark cycle, 20–24 °C, and 60–70% humidity. MDA-MB-231 cells were exposed to PTC-209, palbociclib, or combination of the two inhibitors at 5.0 μM for 72 hrs. Cells were then trypsinized, washed with PBS, and 2 × 10^6^ cells were subcutaneously injected into the right left flank of female nude mice in a 100 μl mixture (1:1 v/v of PBS/matrigel). The animals were monitored twice weekly and tumor volume was measured using caliper. At the end of these experiments, the mice were sacrificed, and the tumors were excised, fixed in 10% buffered formalin, embedded in paraffin, and were subsequently sectioned. Sections were stained with haematoxylin & eosin. Human-specific vimentin staining was used to detect human MDA-MB-231 cells in the xenograft.

### Statistical analysis

Data were presented as mean ± standard error of the mean (S.E.M). Statistical analyses and graphing were performed using GraphPad Prism 8.0 software (GraphPad, San Diego, CA, USA). *P*-values were calculated using the two-tailed *t* test. Data are derived from at least two independent experiments ran in triplicate, unless stated otherwise.

## Results

### Enhanced efficacy of PTC-209 in combination with palbociclib against MDA-MB-231 cells

**Figure 1:**
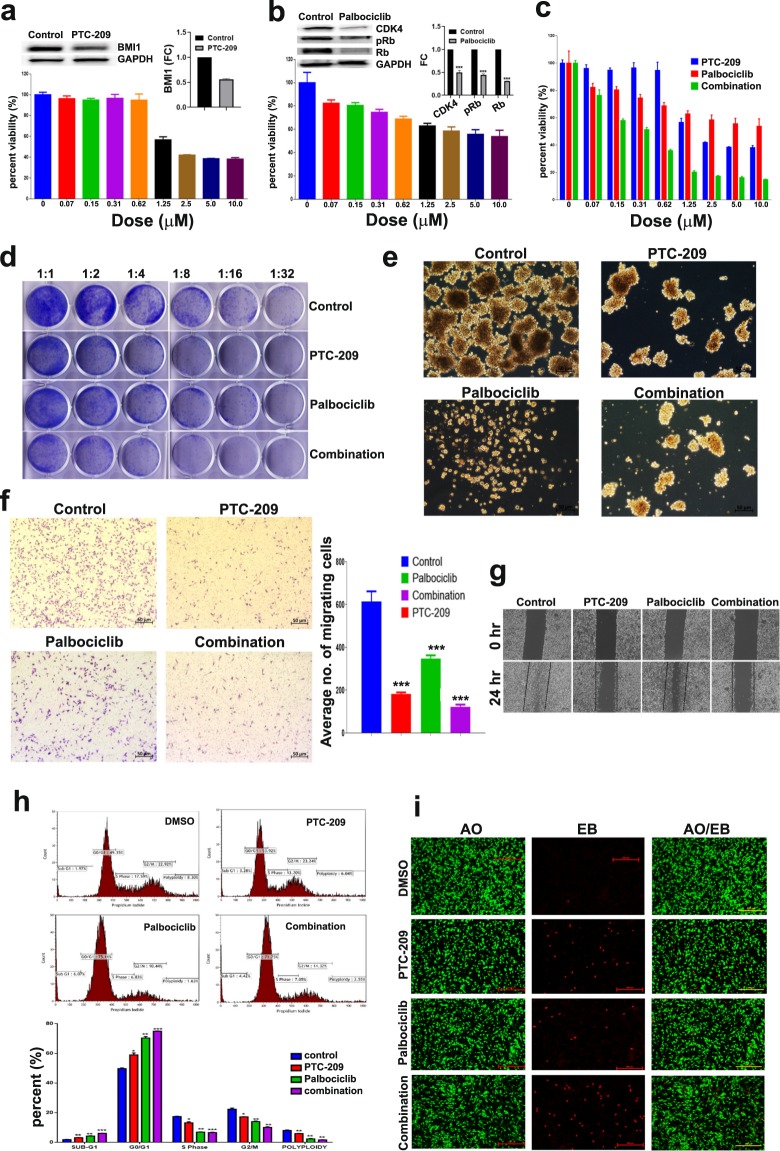
Enhanced efficacy of PTC-209 in combination with palbociclib against MDA-MB-231 cells. MDA-MB-231 cells were treated with the indicated dose of PTC-209 **(a)** and palbociclib **(b)** and cell viability was measured using alamarblue assay on day 4. Suppression of BMI1, CDK4, Rb, and pRb^Ser795^ is shown in upper panels. **(c)** Viability of MDA-MB-231 cells when treated with PTC-209 and palbociclib as single agent or in combination. Data are presented as mean ± S.E.M., n = 6. **(d)** Clonogenic assay showing colony forming capability of MDA-MB-231 treated with PTC-209, palbociclib, or combination of both at 5.0 µM. Plates were stained with Diff-Quik stain set on day 10. Data are representative of two independent experiments for each condition. Effect of PTC-209 and palbociclib, as single agents or in combination on MDA-MB-231 sphere formation **(e)** and cell migration using transwell migration **(f)** and scratch **(g)** assay. The number of migrating cells is indicated. Cell cycle analysis demonstrating the effects of PTC-209 and palbociclib as single agents or in combination on cell cycle distribution in MDA-MB-231 cells (**h**, upper panel). Quantification of cell cycle distribution is shown in the lower panel (n = 3). **(i)** Representative fluorescence images of MDA-MB-231 cells treated with PTC-209 and palbociclib as single agents or in combination (5.0 µM). Cells were stained with acridine orange/ethidium bromide to detect apoptotic (cells with green condensed chromatin) and necrotic (red) cells.

**Figure 2:**
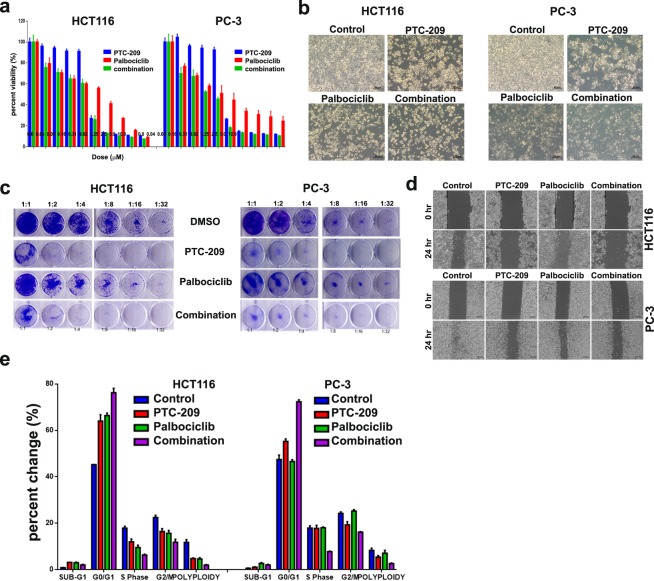
Effects of PTC-209 in combination with palbociclib against HCT116 and PC-3 cancer models. (**a)** Cell viability of HCT116 (left) and PC-3 (right) treated with PTC-209 and palbociclib as single agent or in combination. Data are presented as mean +/− S.E.M., n = 6. **(b)** Representative microscopic images of HCT116 and PC-3 cells under different experimental conditions. **(c)** Clonogenic assay showing colony forming capability of HCT116 (left) cells treated with PTC-209 (0.6 µM), palbociclib (1.25 µM), or combination of both at (0.6 µM + 1.25 µM). Similarly, PC-3 cells (right) were treated PTC-209 (0.6 µM), palbociclib (0.6 µM), or combination of both at (0.6 µM + 0.6 µM). Plates were stained with Diff-Quik stain set on day 10. Wells are representative of two independent experiments for each treatment condition. **(d)** Effect of PTC-209 and palbociclib, as single agents or in combination on HCT116 and PC-3 cell migration using scratch assay at the above mentioned dose. **(e)** Quantification of cell cycle distribution in HCT116 (left) and PC-3 (right) cells treated with PTC-209, palbociclib, or combination of both at the above mentioned dose on day3.

Initially, we performed a dose response curve of MDA-MB-231 cells to PTC-209 treatment as measured by cell viability. This cell line was chosen as a model for TNBC, which is known to have a more aggressive phenotype and limited treatment choices^34,36^. Data presented in Fig. 1a revealed significant inhibition of cell proliferation in a dose-dependent manner (1.25–10 μM) of PTC-209, which was associated with significant reduction in BMI1 protein expression (Fig. 1a, upper panel). PTC-209 doses below 1.25 μM were not effective against MDA-MB-231 cells. Similarly, palbociclib inhibited MDA-MB-231 cell proliferation in a dose-dependent manner (0.07–10 μM, Fig. 1b lower panel), which was associated with significant reduction in CDK4, Rb, and pRb^Serine795^ protein expression (Fig. 1b, upper panel). The combination of the two drugs exerted more inhibitory effects on the growth of MDA-MB-231 cells compared to either drug alone (0.07–10 μM, Fig. 1c). We subsequently assessed the ramifications of PTC-209, palbociclib or the combination of both inhibitors on the clonogenic and sphere formation potential of MDA-MB-231 cells. Data presented in Fig. 1d,e revealed significant inhibition of MDA-MB-231 colony and sphere formation, respectively, which was more profound in the PTC-209 plus palbociclib combination group. Concordantly, combination of PTC-209 and palbociclib was most effective in inhibiting MDA-MB-231 cell migration using the classical boyden chamber (Fig. 1f) and scratch assays (Fig. 1g). In order to gain more insight on the mechanism by which PTC-209 and palbociclib inhibited MDA-MB-231 cell growth, we conducted cell cycle analysis and AO/EB staining under different treatment conditions. Data presented in Fig. 1h,i revealed significant increase in apoptosis (sub-G0) and arrest in the G0/G1 phase of the cells cycle in PTC-209, palbociclib, and the combination treatment group. We subsequently extended our investigations to the HCT116 (colon) and PC-3 (prostate) cancer models, since those two models are known to harbor BMI1^+^ CSC populations^37,38^. Additionally, CDK4 and CDK6 are known to be expressed by both cancer models^39,40^. Combination of PTC-209 and palbociclib exhibited dose-dependent growth inhibitory effects on HCT116 and PC-3 cancer cells (Fig. 2a). Concordantly, combination of PTC-209 and palbociclib was most efficacious in inhibiting the colony formation, as well as the migration of the HCT116 and the PC-3 cancer cells, respectively (Fig. 2b–d). Concordant with data obtained using the MDA-MB-231 model, induction of apoptosis and G1 cell cycle arrest was similarly observed in the HCT116 and the PC-3 models (Fig. 2e) in response to PTC-209 and palbociclib, or combination treatment.

### Multiple dysregulated cellular pathways in PTC-209 treated MDA-MB-231 cells

**Figure 3:**
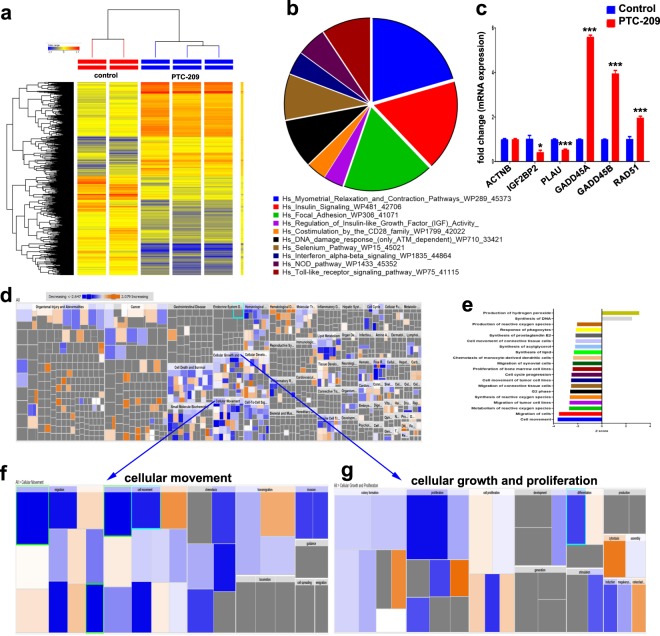
Multiple dysregulated cellular pathways in PTC-209 treated MDA-MB-231 cells. (**a)** Hierarchical clustering of MDA-MB-231 cells treated with PTC-209 compared to vehicle-control treated cells based on differentially expressed mRNA levels using microarray analysis. Each column represents one replica and each row represents a transcript. Expression level of each gene in a single sample is depicted according to the colour scale. **(b)** Pie chart illustrating the distribution of the top 10 pathway designations for the differentially expressed genes in PTC-209 compared to vehicle-control treated MDA-MB-231 cells. The pie size corresponds to the number of matched entities. **(c)** Expression levels of selected genes from the microarray data were validated using qRT-PCR. Data are presented as the means ± S.E.M., n = 6. *p < 0.05; ***p < 0.0005. **(d)** Disease and function heat map depicting enrichment in the indicated functional and disease categories in the differentially expressed transcripts in PTC-209 treated MDA-MB-231 cells based on IPA analysis. Color scale indicates the activation score. **(e)** Bar chart illustrating the significantly enriched functional and disease categories (activation Z score ≤ or ≥2.0). Heat map-illustrating enrichment in cell movement **(f)** or cell growth and proliferation **(g)** functional categories.

To gain more insight into the molecular mechanisms by which PTC-209 affects cancer cellular and functional processes, we performed global mRNA expression profiling on the MDA-MB-231 breast cancer model treated with PTC-209 compared to vehicle-treated control cells. As depicted in Fig. 3a, hierarchical clustering based on differentially expressed mRNA transcripts revealed clear separation between the two treatment groups. A total of 1439 transcripts were upregulated, while 1520 transcripts were downregulated in response to BMI1 inhibition using PTC-209 (2.0 FC, P(corr) < 0.05, Supplementary Table 1). The distribution of the top 10 enriched pathway designations for the differentially expressed genes is shown in Fig. 3b. The expression of selected number of genes from the microarray data was subsequently validated using quantitative reverse transcription PCR (qRT-PCR, Fig. 3c). We subsequently subjected the differentially expressed transcripts in response to PTC-209 treatment into ingenuity pathway analysis software. As shown in Fig. 3d, disease and function analysis revealed remarkable inhibition of cellular movement, growth, and proliferation in PTC-209 treated cells. Top significantly affected disease and function categories (≤2.0 ≤ activation Z score) are shown in Fig. 3e. The cellular movement and cellular growth and proliferation functional categories are depicted in Fig. 3f,g, respectively.

### Palbociclib predominantly target pathways regulating cell cycle and cell proliferation

**Figure 4:**
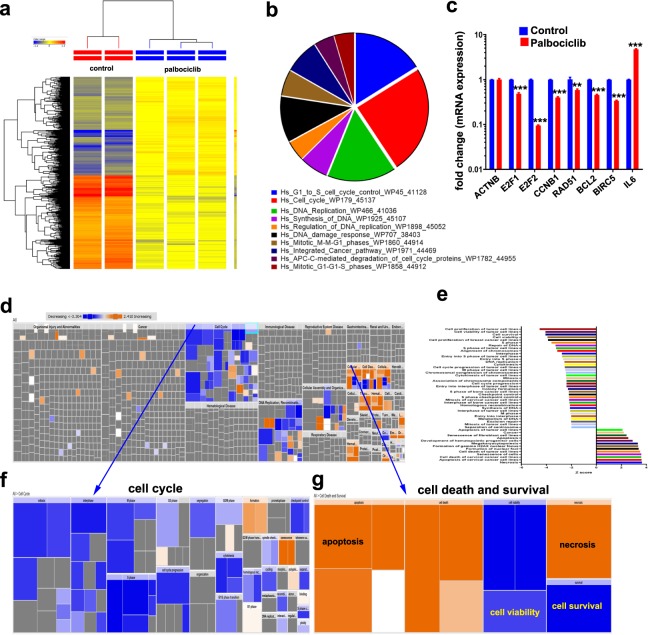
Palbociclib predominantly target pathways regulating cell cycle and cell proliferation. (**a)** Hierarchical clustering of MDA-MB-231 cells treated with palbociclib compared to vehicle-control treated cells based on differentially expressed mRNA levels using microarray analysis. Each column represents one replica and each row represents a transcript. Expression level of each gene in a single sample is depicted according to the colour scale. **(b)** Pie chart illustrating the distribution of the top 10 pathway designations for the differentially expressed genes in palbociclib compared to vehicle-control treated MDA-MB-231 cells. The pie size corresponds to the number of matched entities. **(c)** Expression levels of selected genes from the microarray data were validated using qRT-PCR. Data are presented as the means ± S.E.M., n = 6. **p < 0.01; ***p < 0.001. **(d)** Disease and function heat map depicting enrichment in the indicated functional and disease categories in the differentially expressed transcripts in palbociclib treated MDA-MB-231 cells based on IPA analysis. Colour scale indicates the activation score. **(e)** Bar chart illustrating the significantly enriched functional and disease categories (activation Z score ≤ or ≥2.0). Heat map-illustrating enrichment in the cell cycle **(f)** or cell death and survival **(g)** functional categories.

The signaling pathways affected by palbociclib treatment were subsequently analyzed using global mRNA expression profiling and pathway analysis. As shown in Fig. 4a and Supplementary Table 2, palbociclib treatment led to substantial changes in gene expression of MDA-MB-231 cells. Palbociclib treatment predominantly affected genes involved in cell cycle regulation and DNA replication (Fig. 4b). Validation of selected genes from the microarray data using qRT-PCR is shown in Fig. 4c. The list of differentially expressed genes in palbociclib-treated MDA-MB-231 cells was subsequently subjected to IPA, which revealed significant suppression of genes regulating cell cycle progression, cell survival, while genes involved in promoting cell death were enriched (Fig. 4d). The list of most affected functional categories is shown in Fig. 4e. Cell cycle and cell death and survival functional categories are illustrated in Fig. 4f,g, respectively.

**Figure 5:**
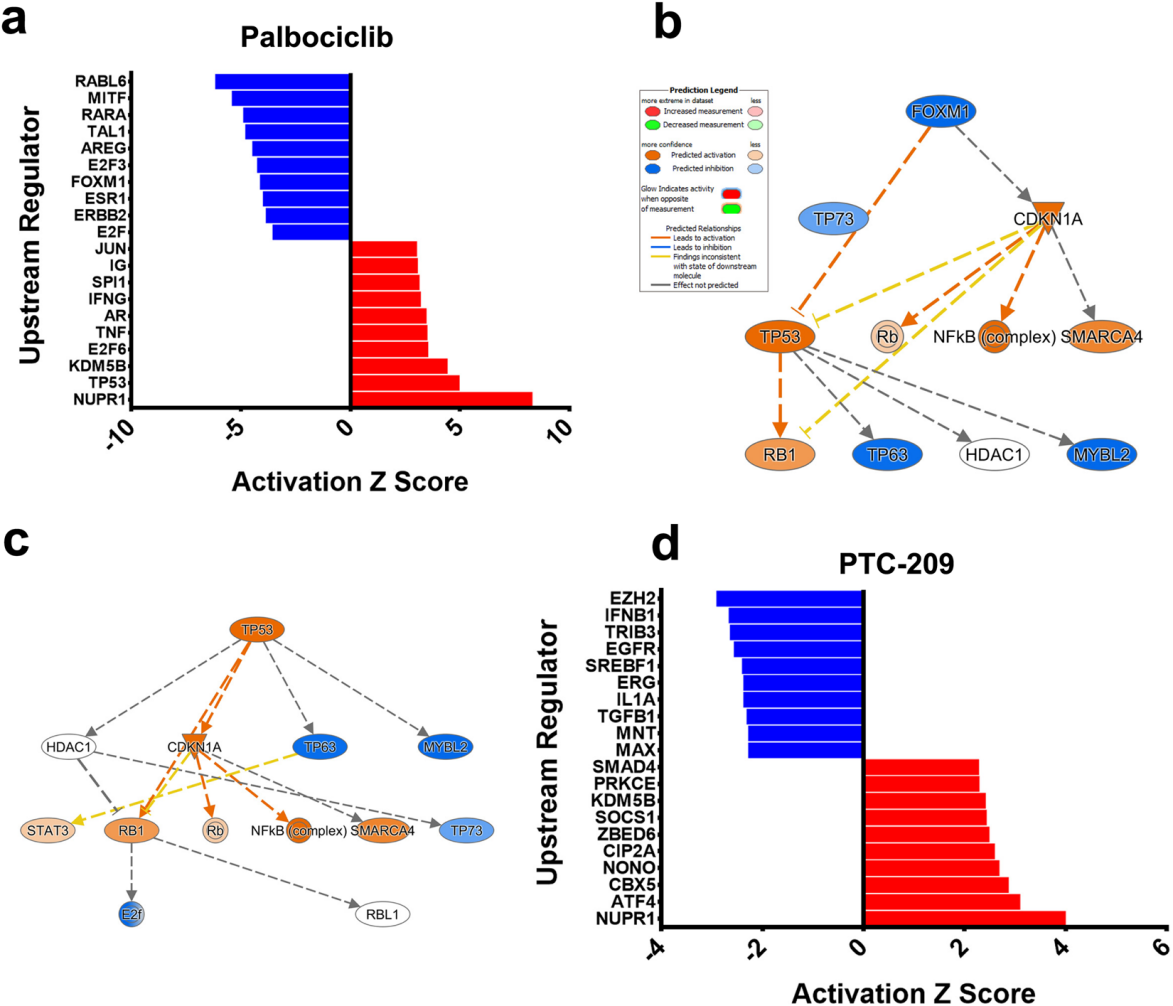
Multiple upstream-regulated networks affected by palbociclib and PTC-209. (**a)** Bar graph plot depicting the top 10 activated and top 10 inhibited upstream networks based on differentially expressed genes in MDA-MB-231 cells treated with palbociclib and IPA analysis. Illustration of the inhibited FOXM1 **(b)** and activated TP53 **(c)** networks is shown. Prediction legend indicated the relationship between different molecules in the network. **(d)** Bar graph plot depicting the top 10 activated and top 10 inhibited upstream networks based on differentially expressed genes in MDA-MB-231 cells treated with PTC-209 and IPA analysis.

Upstream regulator analysis on the differentially expressed genes revealed dramatic effects of palbociclib on several vital networks in cancer cells. Notably, the most inhibited networks by palbociclib were those driven by RABL6, MITF, RARA, TAL1, AREG, E2F3, FOXM1, ESR1, ERBB2, and E2F upstream regulators (Fig. 5a, Supplementary Table 3). Although the expression of several of those upstream regulators was not affected by palbociclib at the transcriptional level, substantial inhibition of downstream targets was observed in our microarray data suggesting inhibition of those networks by palbociclib. Notably, FOXM1 levels were severely downregulated in response to palbociclib treatment (−10.8 FC). The FOXM1 network is illustrated in Fig. 5b. On the other hand, among the top activated networks was NUPR1 and TP53 (Fig. 5a). NUPR1 is a stress-induced chromatin-associated protein, which is necessary for the expression of stress-response genes, including those involved in DNA repair, cell cycle regulation, apoptosis, and autophagy^41^. NUPR1 was upregulated in response to palbociclib treatment (8.8 FC), which is likely as a response to DNA damage and induction of cell death inflicted by palbociclib. Several downstream targets of TP53 were also affected by palbociclib treatment (Supplementary Table 4), which collectively indicated activation of TP53 in palbociclib-treated cells. Illustration of the TP53 network is shown in Fig. 5c. On the other hand, PTC-209 inhibited several networks, including EZH2, IFNB1, TRIB3, EGFR, SREBF1, IL1A, ERG, TGFB1, MAX, and MNT (Fig. 5d, Supplementary Table 5). EZH2 is the core component of the PRC2, which implies disruption of BMI1 by PTC-209 not only affects the PRC1 complex, but it also affects the PRC2 complex. Among the induced networks, we observed activation of NUPR1 network, which is similar to the palbociclib data. Activation of NUPR1 might imply induction of DNA damage and induction of cell death inflicted by PTC-209. Taken together, our data highlighted a number of upstream regulator networks affected by palbociclib and PTC-209 treatment.

### Combination of PTC-209 and palbociclib inhibited MDA-MB-231 tumor formation *in vivo*

**Figure 6:**
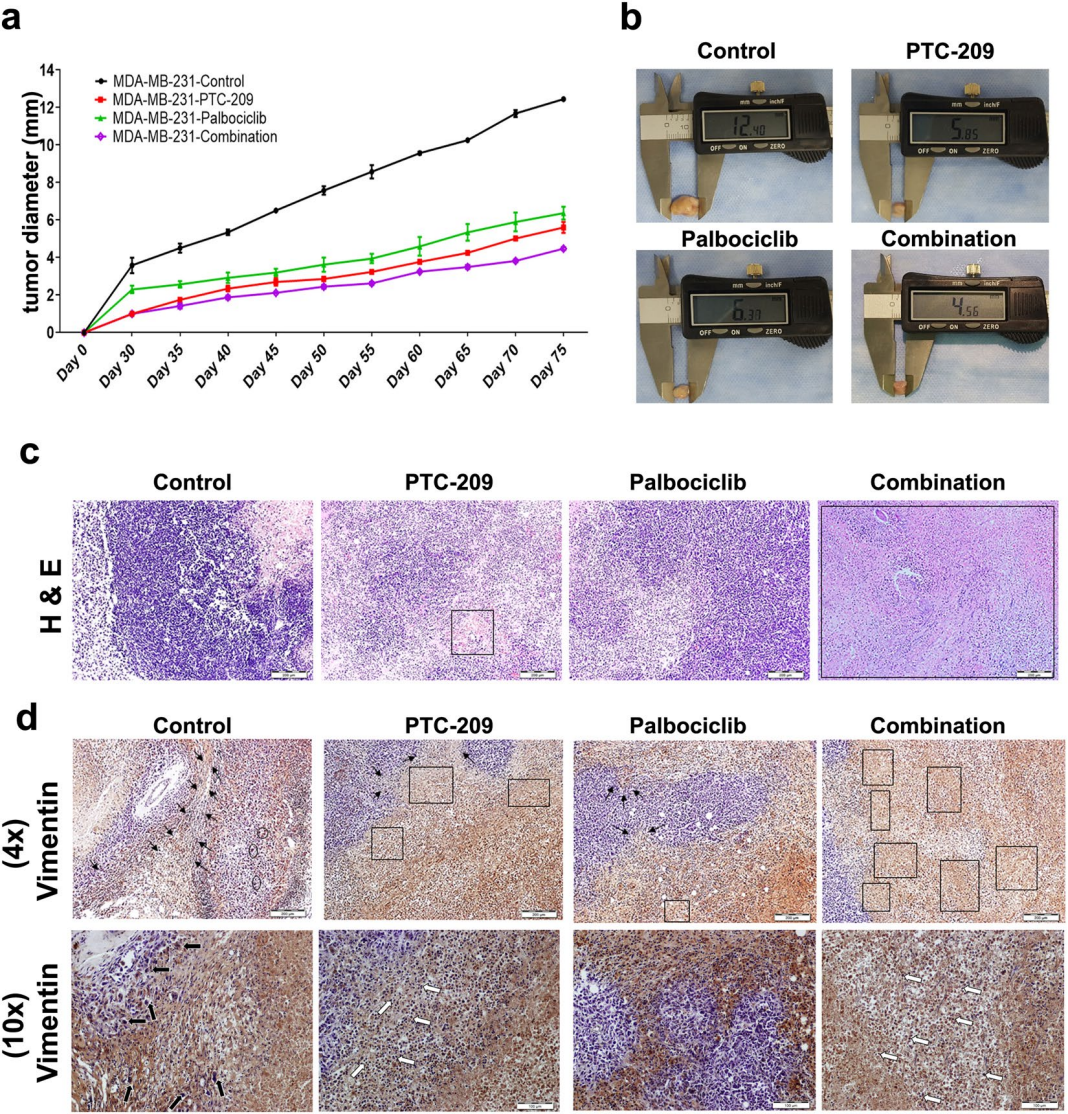
Combination of PTC-209 and palbociclib inhibited MDA-MB-231 tumor formation *in vivo*. (**a)** MDA-MB-231 were treated with PTC-209, palbociclib, or combination of both and were implanted subcutaneously in nude mice as described in materials and methods. Data are presented as mean (tumor diameter) ± S.E.M., n = 2. ROC area under the curve was used to compare different growth curves. **(b)** Representative images of tumors excised from each treatment group at the end of the experiment. **(c**,**d**) Representative histopathological examination of MDA-MB-231 xenograft tumors from each treatment group compared to vehicle-control treatment group. FFPE sections were stained with haematoxylin and eosin stain **(c)** or vimentin **(d)**. (Bar = 200 μm, panel c and d (upper); Bar = 100 μm, panel d (lower)). Black arrow indicates invading cells, square and rectangle indicates necrotic and dead cells and white arrow indicates mitotic events.

To assess the effects of PTC-209, palbociclib, or combination of both on breast cancer tumor formation *in vivo*, MDA-MB-231 were treated with PTC-209, palbociclib, or combination of both for 48 hrs, and subsequently equal number of cells was injected subcutaneously into nude mice. Data presented in Fig. 6a demonstrated significant inhibition of tumor growth by PTC-209 (area under the ROC curve for PTC-209 vs vehicle-treated control: p value = 0.005) and palbociclib (area under the ROC curve for Palbociclib vs vehicle-treated control: p value = 0.014). Interestingly, combination of both drugs was more efficacious in inhibiting MDA-MB-231 tumor growth *in vivo* (area under the ROC curve for PTC-209 plus palbociclib vs vehicle-treated control: p value = 0.0016). Representative images of excised tumors from different treatment groups is shown in Fig. 6b. Histological examination of xenograft tumors revealed a greater degree of cell death and lesser mitotic figures in the combination drug group, while a number of pleomorphic cells were observed in control group (Fig. 6c). Additionally, human specific vimentin staining showed the engraftment of human cells transplant. PTC-209 and Palbociclib treatments restricted the invasiveness of MDA-MB-231 cells, while combination group exhibited the most profound restricted invasion of tumor cells compared to other treatment groups and control (Fig. 6c,d).

## Discussion

Despite recent advances in the development of molecular and cellular-based anti-cancer therapies, chemotherapy remains a main treatment choice for large number of human cancers^42^. However, the evolution of chemoresistence during the treatment process represent a major obstacle for successful chemotherapy^43^. Tumor-initiating cells, also termed cancer stem cells (CSCs), represent a minor subpopulation of cancer cells with intrinsic self-renewal, differentiation and tumor initiation properties, which has also been implicated in drug resistance^44^. In current study, we present the first evidence on the efficacy and elucidated the molecular mechanism by which combination of PTC-209 and palbociclib inhibited the growth of a panel of cancer models (breast: MDA-MB-231; colon: HCT116; prostate: PC-3). Combination of PTC-209 and palbociclib inhibited cell proliferation, sphere and colony formation, cell migration *in vitro*, and tumor formation *in vivo*. Tumor sphere generated *in vitro* is widely acceptable as indicative of self-renewal potential, one of the characteristics of TICs^45^. In current study, we observed remarkable reduction in spheres and colony formation in the combined treatment group; in addition, sphere formed in the combination group were also less compact compared to the control group, which corroborates earlier findings where PTC-209 was shown to reduce sphere formation of biliary tract and colorectal cancer cells^21,46^.

PTC-209 treatment inhibited a number of signaling pathways including those involved in insulin signaling, DNA damage response and wnt/pluoripotency in the MDA-MB-231 breast cancer model. Additionally, PTC-209 inhibited cellular movement and proliferation cellular processes. Metabolic stress has been implicated in the acquisition of stem cell-like characteristics and pharmacological modulation of Wnt signaling pathway disrupting glycolysis in cancer cells^47^. Earlier studies also correlated overexpression of IGF2BP2 in breast cancer and esophageal adenocarcinoma and short patient survival^48^, and as biomarker in several cancer types^35,49^. IGF2BP2 has been implicated in the maintenance of cancer stem cells (CSCs)^50^. Concordantly, blockade of the insulin signaling pathway inhibited the growth and metastasis in several cancer types including breast cancer both *in vitro* and *in vivo*^51,52^. Therefore, our data established targeting the insulin signaling pathway as one potential mechanism by which PTC-209 inhibited tumorigenicity of MDA-MB-231 model.

GADD45, member of the DNA damage response pathway, has been implicated in several signaling pathways and cellular functions, including MAPK signaling, cell cycle regulation, DNA repair, genomic stability, and apoptosis^53,54^. Our data revealed marked increase in the expression of genes involved in DNA damage response such as GADD45A, GADD45B and RAD51 in treated groups. Overexpression of GADD45 family proteins was shown to induce apoptosis in leukemia, lung cancer and HeLa cells^53,55^. Our finding from current study is concordant with our previous data implicating BMI1 in protecting cancer cells from radiation therapy-induced apoptosis^20^. Taken together, our data revealed inhibition of BMI1 using PTC-209 to induce increased DNA damage and to induce apoptosis in cancer cells.

On the other hand, palbociclib treatment of MDA-MB-231 cells lead to substantial changes in gene expression affecting mainly cell cycle progression. In several cancer models, E2F was shown to activate transcriptional programs and to promote cell cycle progression and DNA damage response. Retinoblastoma, a tumor suppressor protein, could prevent abnormal cell growth by suppressing E2F activity and inhibiting the expression of CDK4 and cell cycle progression through the G1/S checkpoint^56^. Present study revealed significant down-regulation of cell cycle and DNA damage response genes, including E2F1, E2F2, RAD51, and CCNB1 in breast cancer cells treated with palbociclib. Our data suggested that suppression of cell cycle and DNA damage response pathways by palbociclib in breast cancer cells leads to cell cycle arrest and apoptosis. In agreement with those data, inhibition of CDK4/CDK6 using PD0332991 suppressed the proliferation of luminal estrogen receptor-positive human breast cancer cell lines and exhibited synergistic effects when combined with tamoxifen and trastuzumab against ER+ and HER2− amplified cell lines, respectively^57^. However, our data is the first to combine PTC-209 and palbociclib against several human cancer models.

Our data provide the first experimental proof on the efficacy of combination therapy targeting BMI1 and cell cycle against breast, colon, and prostate cancer. Combination of PTC-209 and palbociclib inhibited tumor cell proliferation, sphere and colony formation, migration, and *in vivo* tumor formation. Transcriptome and pathway analyses revealed a myriad of signaling pathways and cellular processes affected by PTC-209 and palbociclib. Taken together, our data provide a platform for the future development of combination anti-cancer therapies based on concurrent targeting of self-renewal and cell cycle regulators.

## Supplementary information

supplementary table 1

supplementary table 2

supplementary table 3

supplementary table 4

supplementary table 5
